# Anti-Inflammatory Effect of 1,3,5,7-Tetrahydroxy-8-isoprenylxanthone Isolated from Twigs of *Garcinia esculenta* on Stimulated Macrophage

**DOI:** 10.1155/2015/350564

**Published:** 2015-10-11

**Authors:** Dan-Dan Zhang, Hong Zhang, Yuan-zhi Lao, Rong Wu, Jin-wen Xu, Ferid Murad, Ka Bian, Hong-Xi Xu

**Affiliations:** ^1^Murad Research Center for Modernized Chinese Medicine, Shanghai University of Traditional Chinese Medicine, Cailun Road 1200, Shanghai 201203, China; ^2^School of Pharmacy, Shanghai University of Traditional Chinese Medicine, Cailun Road 1200, Shanghai 201203, China; ^3^Engineering Research Center of Shanghai Colleges for TCM New Drug Discovery, Cailun Road 1200, Shanghai 201203, China; ^4^Department of Biochemistry and Molecular Medicine, George Washington University, I Street, Washington, DC 20037, USA

## Abstract

*Garcinia* Linn. plants having rich natural xanthones and benzophenones with anti-inflammatory activity attracted a great deal of attention to discover and develop them as potential drug candidates. Through screening targeting nitric oxide accumulation in stimulated macrophage, we found that 1,3,5,7-tetrahydroxy-8-isoprenylxanthone (TIE) had potential anti-inflammatory effect. To understand how TIE elicits its anti-inflammatory activity, we uncovered that it significantly inhibits the production of nitric oxide (NO) and prostaglandin E2 (PGE2) in LPS/IFN*γ*-stimulated RAW264.7 cells. In further study, we showed that TIE reduced the expression of inducible nitric oxide synthase (iNOS) and cyclooxygenase-2 (COX-2), two key molecules responsible for the production of NO and PGE2 during inflammation progress. Additionally, TIE also suppressed the expression of inflammatory cytokines IL-6, IL-12, and TNF-*α*. TIE-led suppression in iNOS, COX-2, and cytokines production were probably the consequence of TIE's capability to block ERK and p38MAPK signaling pathway. Moreover, TIE blocked activation of nuclear factor-kappa B (NF-*κ*B) as well as NF-*κ*B regulation of miR155 expression. Our study suggests that TIE may represent as a potential therapeutic agent for the treatment of inflammatory diseases.

## 1. Introduction

Acute inflammatory response represents an initial protective mechanism in the body. However, excessive and chronic inflammation results in severe damage of cells and tissues. Emerging evidences support the hypothesis that chronic inflammation plays a critical role in various pathological conditions, including atherosclerosis, autoimmune disorders, neurodegenerative diseases, and inflammation related various human cancers [1].

Nitric oxide (NO) is a free radical that is synthesized from L-arginine by nitric oxide synthase (NOS). There are three types of NOS: two constitutive NOS, eNOS and nNOS, and one inducible NOS (iNOS). Constitutive NOS generate nanomolar concentration of NO and are known to mediate various physiological functions. Contrarily, iNOS produces NO at the level of micromolar concentration that often results in pathological consequences such as chronic inflammation [2]. PGE2 are synthesized from arachidonic acid by cyclooxygenase (COX) during the inflammatory reaction. Two COX isoenzymes are known as COX-1 and COX-2. COX-1 is expressed constitutively in most cells and involved in homeostasis, whereas COX-2 is not produced in normal tissues until being induced by chemical and physical stimulations and enhanced by oncogenes, growth factors, and cytokines [3].

LPS triggers a series of signal transduction events which lead to the activation of NF-*κ*B, mitogen-activated protein kinase (MAPK) signaling pathway, and differential expression of miRNAs that contribute to the inflammatory response [4].

NF-*κ*B is an essential transcription factor that regulates proinflammatory gene expression such as iNOS, COX-2, and interleukin-6 (IL-6) [5]. In mammals, three major MAPKs subfamilies have been described such as ERK, JNK, and p38 MAPK. Secretion of several macrophage factors such as IL-6 and NO requires MAPK activity [6]. miR155 represents a typical multifunctional miRNA and contributed to the progressive inflammatory diseases that expression in macrophages was correlated positively with proinflammatory cytokine expression [7, 8].

There are some cross talks between these classical inflammatory pathways. MAPKs can be activated by Toll-like receptor 4 (TLR4) leading to the activation of nuclear translocation of NF-*κ*B and finally initiate proinflammatory responses [9]. NF-*κ*B is activated by phosphorylation of I*κ*B*α* via activation of MAPKs and then migrates into the nucleus and activates the expression of inflammatory cytokines and mediators [10]. miR155 induction requires NF-*κ*B signaling to upregulate fos/jun transcription factors during the responses to infection [11].

*Garcinia esculenta *Y. H. Li (Clusiaceae) is one of* Garcinia* Linn. species, the fruit of which is edible and juicy with sweet and sour taste; meanwhile it is a well-known Chinese traditional medicine with multiple pharmacological functions in treating inflammation and tumor, distributed in the western and northwestern part of Yunnan province in China [12]. However, mechanisms associated with its anti-inflammatory effect are not clear. During the previous course of bioassay-guided screening compounds from twig of* Garcinia esculenta *Y. H. Li, we found 1,3,5,7-tetrahydroxy-8-isoprenylxanthone (TIE) suppressed NO accumulation in stimulated macrophage [13]. In the present paper, we further study its mechanism on LPS/IFN*γ*-induced inflammatory responses in murine macrophages RAW264.7 cells.

## 2. Materials and Methods

### 2.1. Materials and Reagents

**Figure 1:**
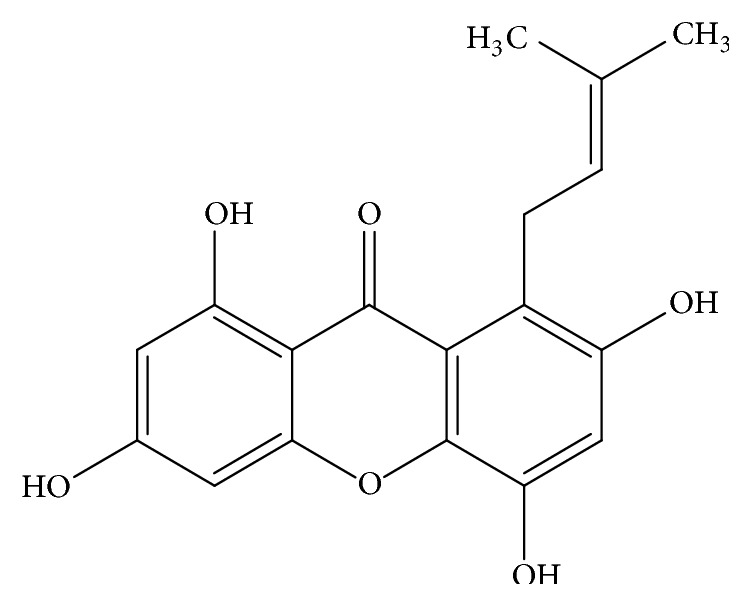
Chemical structure of TIE.

TIE (molecular weight 328 Da) was isolated from twig of* Garcinia esculenta *Y. H. Li (Clusiaceae), and the collection, identification, and specimen restoration were described in the previous report [13]. The chemical structure of TIE is shown in Figure 1. The purity of TIE was detected by HPLC and the results suggested a purity of above 98%.

Murine recombinant IFN*γ* and NF-*κ*B p50/p65 EZ-TFA transcription factor assay kit were purchased from Millipore (Bedford, MA, USA); mouse PGE2 and IL-6 ELISA kit were purchased from Cayman Chemical Company (Ann Arbor, MI, USA); lipopolysaccharide (LPS;* Escherichia coli* O111:B4), dimethyl sulfonamide (DMSO),* N*-(1-naphthyl)-enthylendiaminedihydrochloride, 3-(4,5-dimethylthiazol-2-yl)-2,5-diphenyltetrazoleum (MTT), and L-N^6^-(1-iminoethyl)lysine hydrochloride (L-NIL, iNOS selective inhibitor) were obtained from Sigma Chemical Co. (St. Louis, MO, USA); RPMI-1640 was purchased from Gibco Invitrogen Corporation (Grand Island, NY, USA); fetal bovine serum (FBS) was purchased from Hyclone (Logan, UT, USA); TRIzol Reagent, lipofectamine TM 2000, Reverse Transcription Kit, and SYBR Green PCR Master Mix regents were obtained from Invitrogen (Carlsbad, CA, USA). The promoter-luciferase plasmid for NF-*κ*B was kindly provided by Professor Jin-wen Xu; Dual Luciferase Reporter reagents were purchased from Promega (Madison, WI, USA). Antibodies used in this study include the following: iNOS monoclonal antibody from Abcam (Cambridge, MA, USA); p-p38, T-p38, p-JNK, T-JNK, p-ERK1/2, and T-ERK1/2 and *β*-actin antibodies from Cell Signaling Technology (Beverly, MA, USA); TaqMan MicroRNA Reverse Transcription Kit and TaqMan Universal Master Mix purchased from Applied Biosystems (Waltham, MA, USA); ECL regent kit purchased from GE Healthcare Life Sciences (Buckinghamshire, UK).

### 2.2. Cell Culture

RAW264.7 cells were originally obtained from the American Tissue Culture Collection. Cells were maintained in RPMI 1640 medium supplemented with 10% FBS at 37°C in a humidified 5% CO_2_ atmosphere.

### 2.3. Assay for Cell Viability

Cell viability was assessed by MTT assay. RAW264.7 cells were cultured in a 96-well plate (5000 cells/well) in an incubator at 37°C, 5% CO_2_, and 95% humidity. TIE was dissolved in DMSO and added after dilution with culture media to 3.125, 6.25, 12.5, and 25 *μ*M at final concentration of DMSO never exceeding 0.1%. The cells were incubated with 10 *μ*L MTT (5 mg/mL in phosphate-buffered saline, pH = 7.4) for 4 h at 37°C and discarded the supernatant followed by adding 150 *μ*L DMSO. Absorbance was measured at 490 nm in a microplate reader (Molecular Devices, Sunnyvale, CA). The absorbance of control (untreated) cells was considered as 100% of viability.

### 2.4. Measurement of NO Production

RAW264.7 cells were plated in a 96-well plate (1 × 10^5^ cells/well) overnight, followed by the addition of 10 U/mL IFN*γ* and 100 ng/mL LPS for 24 h in the presence or absence of different dosage of TIE with increasing concentration at 3.125, 6.25, 12.5, and 25 *μ*M, and L-NIL (50 *μ*M), a selected iNOS inhibitor. To analyze NO production, 100 *μ*L of supernatant was incubated with equal volume of Griess solution (1% sulfanilamide in 5% phosphoric acid and 1%  *α*-naphthylamine in distilled water) at room temperature for 10 min and absorbance was then read at 540 nm. Since NO content was reflected by the amount of nitrite, a calibration curve was generated using sodium nitrite. The amount of nitrite in the supernatants was calculated based on the calibration curve. The percentage inhibition of NO production is evaluated using the formula {1-[(nitrite amount of fraction-treated)/(nitrite amount of vehicle)]} × 100.

### 2.5. Detection of PGE2 and IL-6 in Supernatant

Inhibitory effects of TIE on the PGE2 and cytokine IL-6 production from LPS plus IFN*γ* treated RAW264.7 cells were detected by sandwich ELISA KIT according to the manufacturer's instruction. After incubation with different dosage of TIE and stimulation with LPS plus IFN*γ* on RAW264.7 cells for 24 h, supernatants were harvested and assayed for PGE2 and IL-6. Results of three independent experiments were used for statistical analysis. L-NIL (50 *μ*M) was used as the positive control.

### 2.6. RNA Isolation and Quantitative qRT-PCR

Total RNA was extracted using TRIzol Reagent according to manufacturer's instruction. The concentration and integrity of purified RNA were measured by absorption of light at 260 and 280 nm (A260/280). From each sample, 2.0 *μ*g of total RNA was then reverse transcribed to single-stranded cDNA by Invitrogen Reverse Transcription Reagents. Then qPCR analyses were performed on the aliquots of the cDNA preparations with SYBR Green PCR Master Mix to detect quantitatively the gene expression of iNOS, COX-2, IL-6, IL-12p35, IL-12p40, TNF-*α*, TBK1, and *β*-actin (an internal standard) using Applied Biosystems 7500HT Fast Real-Time PCR System (Applied Biosystems Inc., Foster City, CA, USA). The 2^−ΔΔCT^ method was utilized to analyze the fold increase. The primers used (Sangon, shanghai, China) are listed in Table 1.

### 2.7. TaqMan MicroRNA Real-Time RT-PCR Assays

The reactions were set according to the manufacturer's protocol. Briefly, total RNA was purified by TRIzol. For each reaction, 10 ng of total RNA was used for reverse transcription using TaqMan MicroRNA Reverse Transcription Kit and reverse transcription primers for mmu-miR155 and the housekeeping gene RNU6B. Real-time PCR quantification was performed using TaqMan PCR primers and TaqMan Universal Master Mix using the following conditions: 16°C for 30 min, 42°C for 30 min, and 85°C for 5 min on Applied Biosystems 7500HT Fast Real-Time PCR System. The samples were measured in triplicate cases. RNU6B endogenous control was used for normalization, and expression levels were presented as 2^−ΔΔCT^ with standard deviation.

### 2.8. Transient Transfection and Dual Luciferase Reporter Assay

For the reporter assay, briefly, cells were seeded into 24-well plates at a density of 5 × 10^5^ cells/well in 500 *μ*L of DMEM without antibiotics and incubated overnight. The cells in each well were transiently transfected with NF-*κ*B luciferase reporter construct and internal control Renilla luciferase vector using lipofectamine TM 2000 reagent according to the manufacturer's procedures. Five hours after transfection, the cells were washed with phosphate-buffered saline and then supplied with fresh medium with FBS and treated with TIE (3.125, 6.25, 12.5, and 25 *μ*M) and L-NIL (50 *μ*M) as the positive control for 1 h before stimulation with LPS and IFN*γ* for 18 h. Subsequently, luciferase activities were measured in cell lysates placed in opaque 96-well plates using Dual Luciferase Reporter reagents following manufacturer's instruction. Luciferase activity was normalized to transfection efficiency as monitored by Renilla luciferase expression. The level of luciferase activity was determined compared to control cells with no stimulation.

### 2.9. The DNA-Binding Activity of NF-*κ*B Assay

The DNA-binding activity of NF-*κ*B in nuclear extracts was measured using the NF-*κ*B p50/p65 EZ-TFA transcription factor assay kit according to the manufacturer's instructions. Briefly, the cells were pretreated with different-concentrations of TIE and stimulated for 30 min with LPS plus IFN*γ*. The nuclear protein extracts of each sample were added to each well after measuring its concentration. The plates were incubated for 1 h at room temperature. After washing each well with wash buffer, 100 *μ*L of diluted NF-*κ*B antibody was added to each well, and then the plates were incubated further for 1 h. After washing wells with wash buffer, 100 *μ*L of diluted HRP-conjugated antibody was added to each well, followed by 1 h incubation. 100 *μ*L of developing solution was added to each well for 5 min, followed by the addition of stop solution. Finally, the absorbance of each sample at 450 nm was determined with a spectrophotometer within 5 min, and the final p50/p65 binding activity of each treatment group was normalization by protein concentration.

### 2.10. Protein Extraction and Western Blotting Analysis

Protein samples (25–50 *μ*g/mL) were mixed with loading buffer, boiled for 5 min, and then separated through 7.5% or 12% sodium dodecyl sulfate-polyacrylamide gel electrophoresis (SDS-PAGE). Proteins were transferred to nylon membranes by electrophoretic transfer. The membranes were blocked in 10% nonfat dry milk (1 h), rinsed, and incubated with primary antibodies in TBST overnight at 4°C. Primary antibody was removed, membranes were washed three times in TBST, and peroxidase-labeled secondary antibody was added for 1 h at room temperature. Following three washes in TBST, bands were visualized by ECL regent kit and exposure to X-ray film.

### 2.11. Statistical Analysis

The results are presented as means ± standard error of the mean (SEM). Student's test was used to analyze the difference between treated and untreated groups. Statistically significant differences between multiple groups were calculated by the application of an analysis of variance (ANOVA) test. *P* < 0.05 was considered statistically significant.

## 3. Results

### 3.1. Concentration-Dependent Inhibition of TIE on LPS/IFN*γ*-Induced NO and PGE2 Production with Nontoxic Effects

**Figure 2:**
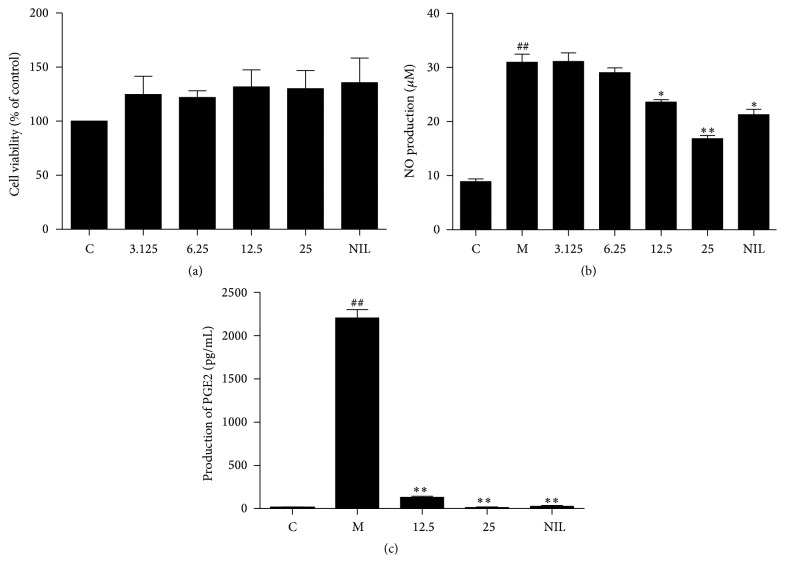
Effect of TIE on NO and PGE2 production in LPS/IFN*γ*-stimulated RAW264.7 macrophages. (a) RAW264.7 cells were treated with TIE (3.125–25 *μ*M) or 50 *μ*M L-NIL for 24 h. Cell viability was measured by MTT assay. Changes in survival are represented as percentages of the control group. (b) Cells were plated at a density of 1 × 10^5^ cells/well in a 96-well plate and allowed to attach overnight. Cells were treated with the various concentrations of TIE (3.125–25 *μ*M) or 50 *μ*M L-NIL and incubated with LPS (100 ng/mL) and IFN*γ* (10 U/mL) in fresh FBS-free medium for 24 h. The nitrite production was measured by the Griess reaction. (c) Cells were treated with the indicated concentrations of TIE (12.5, 25 *μ*M) and incubated with LPS (100 ng/mL) and IFN*γ* (10 U/mL) in fresh FBS-free medium for 24 h. The PGE2 concentration in cell supernatant was determined by ELISA kit. The values were presented as mean ± SEM of three independent experiments. ^##^ *P* < 0.01; ^#^ *P* < 0.05 versus control group; ^*∗∗*^ *P* < 0.01; ^*∗*^ *P* < 0.05 versus model group.

To determine the effects of TIE on cell viability, RAW264.7 cells were initially seeded in 96-well plates followed by different treatments. Results of the MTT assay after 24 h treatment indicated that none of the treatments with TIE at the different concentrations from 3.125 to 25 *μ*M was toxic as compared to the untreated cells, as well as positive control L-NIL at 50 *μ*M (*P* > 0.05) (Figure 2(a)).

Since TIE has been shown to exhibit inhibition of NO production in our previous screening, increasing concentration of TIE (3.125, 6.25, 12.5, and 25 *μ*M) was tested on RAW264.7 cells with and without stimulation. The result shows that 24 h inflammatory factors treatment triggered 30-fold increase of nitrite concentration, a biomarker of NO production. Application of TIE dose-dependently attenuated NO production while treatments with TIE without LPS stimulation had a mild inhibitory effect on NO production (data not shown), with a maximum effect of 62.51% NO reduction with 25 *μ*M TIE (Figure 2(b)). TIE also reduced PGE2 production in a concentration-dependent manner after treatment of 24 h (Figure 2(c)).

These results suggest that TIE selectively inhibits NO and PGE2 production. These results also indicate that the inhibitory effect of TIE on NO and PGE2 production in LPS/IFN*γ*-stimulated cells is not caused by cellular toxicity.

### 3.2. TIE Treatment Prevents LPS/IFN*γ*-Induced iNOS and COX-2 Expression at mRNA and Protein Level

**Figure 3:**
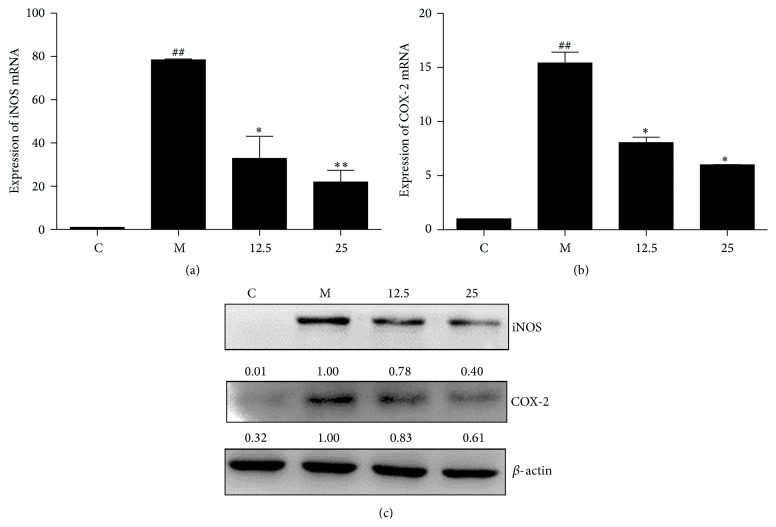
Effect of TIE on iNOS and COX-2 expression in LPS/IFN*γ*-stimulated RAW264.7 cells. (a, b) RAW264.7 cells were plated at a density of 1 × 10^6^ cells in 30 mm dish overnight and treated with varying concentrations of TIE with treatment of LPS (100 ng/mL) and IFN*γ* (10 U/mL) for 4 h. Total RNA was isolated and subjected to qRT-PCR. *β*-actin mRNA was used as an internal control for standardization. (c) RAW264.7 cells were plated at a density of 1 × 10^6^ cells in 30 mm dish overnight. TIE was added with the treatment of IFN*γ* (10 U/mL) plus LPS (100 ng/mL) for 6 h. Whole cell lysates were prepared and subjected to Western blotting. *β*-actin protein was used as an internal control for standardization. The data shown are representative of three independent experiments. ^##^ *P* < 0.01,  ^#^ *P* < 0.05 versus control group; ^*∗∗*^ *P* < 0.01, ^*∗*^ *P* < 0.05 versus model group.

Since TIE can effectively reduce LPS/IFN*γ*-induced NO production, the fact that iNOS is responsible for LPS/IFN*γ*-induced NO production indicates that TIE might block NO production by decreasing the amount of iNOS. We hypothesized that TIE might also possess potent inhibition of iNOS expression, since iNOS always regulated by NF-*κ*B at transcription level [14]. We tested this possibility by determining the effect of TIE on iNOS mRNA and protein in LPS/IFN*γ*-stimulated RAW264.7 cells with the aid of quantitative RT-PCR (qRT-PCR) and Western blot analysis. Results showed that LPS/IFN*γ* stimulation elevated the mRNA and protein level of iNOS, and TIE pretreatment diminished LPS/IFN*γ*-induced iNOS gene and protein expression in RAW264.7 cells. TIE was found to decrease the levels of COX-2 mRNA and protein too (Figure 3).

These results suggested that TIE might significantly suppress LPS-induced PGE2 via inhibiting COX-2 expression at the transcriptional level.

### 3.3. Secretion and Expression of Inflammatory Cytokines Are Suppressed by TIE

The effects of TIE on the secretion and expression of proinflammatory cytokines including IL-6, IL-12, and TNF-*α* were investigated by ELISA kit and qRT-PCR, respectively.

TNF-*α* and IL-6 are potent proinflammatory cytokines induced during inflammation progress, accompanied with interleukin-12 (IL-12) playing the essential role in immune defense against infection [15, 16].

**Figure 4:**
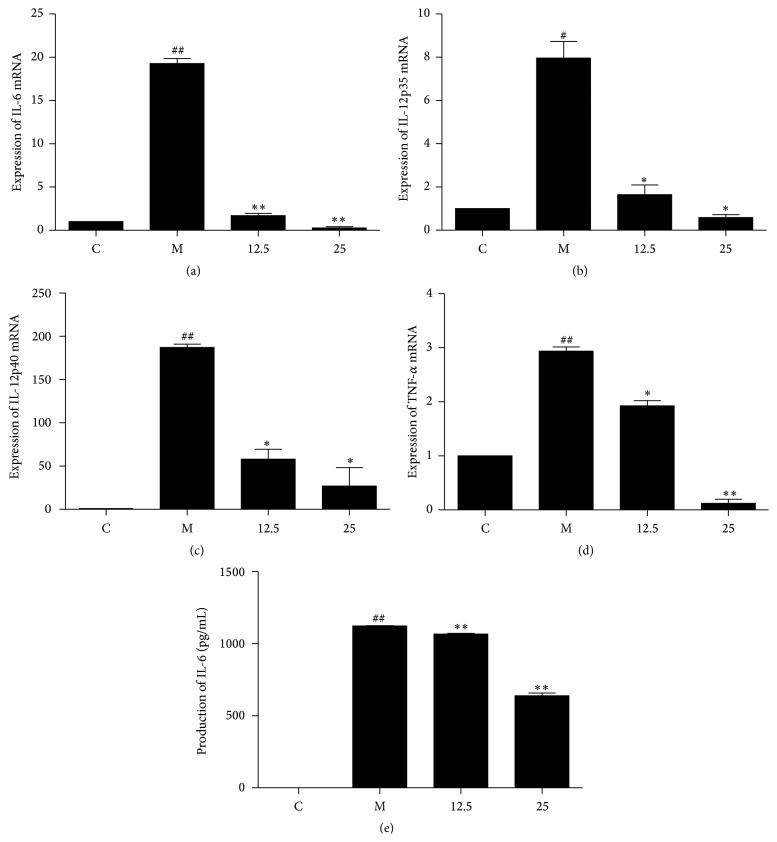
Effect of TIE on proinflammatory cytokines in LPS/IFN*γ*-stimulated RAW264.7 cells. (a, b, c) RAW264.7 cells (1 × 10^6^ cells/dish) were treated with varying doses of TIE with IFN*γ* (10 U/mL) plus LPS (100 ng/mL) for 4 h. Total RNA was isolated and subjected to qRT-PCR to determine the level of IL-6 and IL-12p35/p40 mRNA. (d, e) RAW264.7 cells were treated with IFN*γ* (10 U/mL) plus LPS (100 ng/mL) in the presence of varying concentrations of TIE for 24 h. Total RNA was isolated and subjected to qRT-PCR to determine the level of TNF-*α* mRNA. Conditioned media were collected and subjected to ELISA to determine the amount of IL-6. The values (means ± SEM) were obtained from three independent experiments. ^##^ *P* < 0.01, ^#^ *P* < 0.05 versus control group; ^*∗∗*^ *P* < 0.01, ^*∗*^ *P* < 0.05 versus model group.

Under stimulation of LPS plus IFN*γ* for 4 h, the mRNA levels from proinflammatory genes IL-6, IL-12p35, and p40 were highly induced and TNF-*α* enhanced its expression after 24 H stimulation. Treatment of cells with TIE significantly decreased the expression of IL-6, IL-12p35/p40, and TNF-*α* (Figures 4(a), 4(b), 4(c), and 4(d)).

LPS/IFN*γ* stimulation increased not only IL-6 expression, but also the secretion of IL-6. Coincubation of TIE and proinflammation stimulation for 24 h showed the strong suppression of this proinflammatory cytokine in cell supernatant (Figure 4(e)).

These data showed that TIE maybe interfere in the transcription level and protein secretion progress of IL-6. Inflammatory cytokines IL-12p35/p40 and TNF-*α* also can be reduced by TIE.

### 3.4. TIE Inhibits Induced NF-*κ*B Luciferase Activity via Suppression Nuclear Translocation of the p65 and p50 Subunits

NF-*κ*B plays a pivotal role in regulation of the expression of iNOS, COX-2, and inflammatory cytokines such as IL-6 and IL-12 [5]. To investigate the underlying mechanism of the inhibition of TIE on iNOS and COX-2 expression in stimulated cells, luciferase reporter assay was used to explore the effects of TIE on NF-*κ*B-dependent reporter gene expression following proinflammatory treatment. RAW264.7 cells were transiently cotransfected with a pNF-*κ*B reporter vector.

Cells were incubated with TIE (3.125, 6.25, 12.5, and 25 *μ*M) and LPS plus IFN*γ* for 18 h.

**Figure 5:**
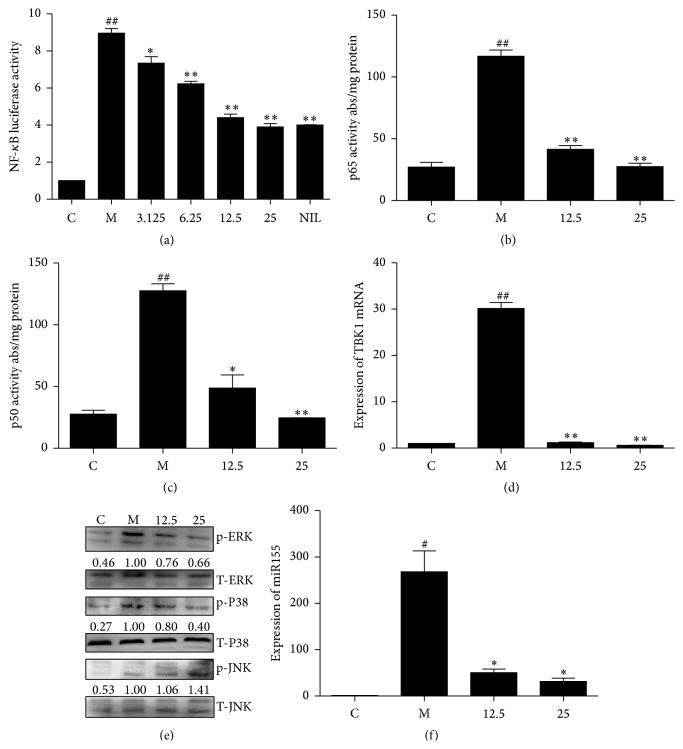
Effect of TIE on NF-*κ*B, MAPK activation, and miR155 expression in LPS/IFN*γ*-stimulated RAW264.7 cells. (a) RAW264.7 cells were transiently cotransfected with a pNF-*κ*B reporter vector. Cells were incubated with TIE (3.125, 6.25, 12.5, and 25 *μ*M) and L-NIL (50 *μ*M) for 1 h before stimulation with LPS and IFN*γ* for 18 h. Luciferase activities were measured by Dual Luciferase Reporter reagents following manufacturer's instruction. Luciferase activity was normalized to transfection efficiency as monitored by Renilla luciferase expression. (b, c) DNA-binding activity of p65 and p50 proteins in nuclear extracts was assessed using NF-*κ*Bp50/p65 EZ-TFA transcription factor assay. Absorbance was measured at 450 nm in a microplate spectrophotometer. Results were normalized to absorbance/mg protein. (d) RAW264.7 cells (1 × 10^6^ cells/dish) were treated with varying doses of TIE with IFN*γ* (10 U/mL) plus LPS (100 ng/mL) for 4 h. Total RNA was isolated and subjected to qRT-PCR to determine the level of TBK1 mRNA. (e) RAW264.7 cells were plated at a density of 1 × 10^6^ cells/well in 30 mm dish overnight. TIE was added to cells followed by 30 min stimulation of IFN*γ* (10 U/mL) plus LPS (100 ng/mL). Whole cell lysates were prepared and subjected to Western blotting. The ratios of immunointensity of p-ERK1/2, p-JNK, and p-p38 were calculated, respectively. Total ERK1/2, JNK, and p38 (T-ERK1/2, T-JNK, and T-p38) were used as a control of the protein amount in the same samples. Data shown are the representative of three independent experiments. (f) The cells were stimulated by IFN*γ* (10 U/mL) plus LPS (100 ng/mL) with or without 12.5 and 25 *μ*M concentrations of TIE for 24 h. Total RNA was isolated and the expression of miR155 was determined by qRT-PCR. RNU6B was used here as an endogenous control. The data represent the mean ± SD of triplicate experiments. ^##^ *P* < 0.01,  ^#^ *P* < 0.05 versus control group; ^*∗∗*^ *P* < 0.01,  ^*∗*^ *P* < 0.05 versus model group.

The results indicated that LPS/IFN*γ* treatment for 18 h induced NF-*κ*B reporter activity almost 9-fold, and TIE reduced the expression of NF-*κ*B luciferase in a concentration-dependent manner (Figure 5(a)), with a maximum effect of a 63.50% reduction when the cells were treated with 25 *μ*M TIE, which was similar to the results of the positive control L-NIL at the concentration of 50 *μ*M.

Because p65 and p50 are the major subunits of the NF-*κ*B heterodimer, the translocation of p65 and p50 subunits from the cytoplasm to the nucleus after being released from I*κ*B*α* was well investigated. As shown in Figures 5(b) and 5(c), the concentrations of p65 and p50 subunits were increased in the nucleus after LPS/IFN*γ* treatment; pretreatment with TIE reversed these trends.

TBK1 belongs to I*κ*B kinase (IKK) family that can active IRF3/IRF7 and NF-*κ*B pathway to regulate inflammatory responses in macrophages [17, 18]. So we also detected the expression of TBK1; the result showed TBK1 was influenced by TIE (Figure 5(d)).

Taken together, these findings confirmed that TIE suppressed the expression of iNOS, COX-2, and cytokines at least in part via NF-*κ*B-dependent mechanism.

### 3.5. MAPK Signaling Pathways Are the Target of TIE-Mediated Inhibition

LPS induction of cytokine expression occurs via activation of MAPK and key protein phosphorylation following binding to TLR4 [4]. MAPKs play an important role in the transcriptional regulation of LPS-induced expression of iNOS and COX-2 via activation of the transcription factor NF-*κ*B [8]. To determine whether the anti-inflammatory effects of TIE are mediated through the MAPK inactivation, Western blots were performed to analyze the levels of p-ERK, JNK, and p38 in RAW246.7 cells. LPS/IFN*γ* stimulation (30 min) evocated significant increases in the levels of phosphorylated ERK, JNK, and p38 in RAW246.7 cells. Coincubation of TIE markedly inhibited the extent of ERK and p38 phosphorylation; meanwhile the amounts of nonphosphorylated p38 and ERK1/2 were apparently unaffected by LPS or LPS plus TIE treatment (Figure 5(e) and supplementary data in the Supplementary Material available online at http://dx.doi.org/10.1155/2015/350564). These results suggest that TIE blocks inflammatory responses by the combination of blocking NF-*κ*B, ERK, and p38 activation and slightly enhanced phosphorylation of JNK (p-JNK) (*P* > 0.05). These results suggested that the anti-inflammatory activity of TIE was mediated by inhibition of the LPS-induced phosphorylation of p38 and ERK1/2. We found that TIE attenuated the activation of p38, rather than that of ERK by stimulation.

These results indicated that the inhibitory effects of TIE on NO and PGE2 were mediated partly via the downstream MAPKs pathway.

### 3.6. miR155 Repression Was Involved in TIE Anti-Inflammatory Effects

MAPKs and NF-*κ*B, two downstream pathways of TLR4 signalling, were shown to positively regulate the expression of miR155 [14]. As miR155 play important roles in the innate immune response and inflammation, we evaluated the effect of TIE on the expression of miR155 by TaqMan MicroRNA assay. As shown in Figure 5(f), miR155 also dramatically increased after 24 h stimulation, and TIE reduced LPS plus IFN*γ*-induced miR155 expression by 85.87%–90.77% from the dosage from 12.5 to 25 *μ*M.

This result showed that miR155 repression was involved in TIE anti-inflammatory effects of NO, PGE2, and IL-6 production, as a complementary regulation at an epigenetics level.

## 4. Discussion

Natural production has been used as medicine for treating a wide variety of disorders including acute and chronic inflammation.* Garcinia esculenta *Y. H. Li (Clusiaceae) is a well-known herb in treating inflammation and tumor for hundreds of years according to Chinese traditional medicine though material basis and its mechanism of anti-inflammation are still unclear.

With the help of the activity guiding extraction and separation, we caught some compounds isolated from* Garcinia esculenta *Y. H. Li (Clusiaceae) [13]. Among these compounds, TIE had the strongest capability of inhibition of NO production on stimulated macrophage RAW264.7 during the screening procedure, which indicated the potential of its anti-inflammatory effect* in vitro*.

However the mechanisms of anti-inflammatory effects of TIE have not been delineated yet. Thus, our study aimed to elucidate the mechanisms underlying the anti-inflammatory effects of TIE.

It is well known that the overproduction of NO and PGE2 by iNOS and COX-2 plays a critical role in the regulation of the inflammatory process. Therefore, to study the suppression of NO and PGE2 by iNOS and COX-2 is very important in the development of anti-inflammatory agents [19, 20]. Selective iNOS inhibitor showed the anti-inflammatory and antitumor properties in animal models [21, 22]. Here, we demonstrated that TIE can dose-dependently inhibit LPS plus INF-induced NO production in RAW264.7 macrophages, as well as suppression of PGE2 (Figure 2). Consistent with these findings, TIE also suppressed induced expression of iNOS and COX-2 at the mRNA and protein levels in RAW264.7 macrophages (Figure 3), which suggested that TIE-induced reduction of NO and PGE2 may be due to transcriptional suppression of iNOS and COX-2 genes.

Induction of iNOS is often accompanied with upregulation proinflammatory cytokines in macrophages [23]. IL-6, IL-12, and TNF-*α* play a critical role in innate immune responses and it is the principal mediator in response to LPS stimulated tissue injury and shock [24]. Therefore, we also investigated the effect of TIE on LPS plus IFN*γ*-inducible proinflammatory cytokines expression and secretion. TIE can also diminish the expression and secretion of IL-6 as well as gene expression of IL-12 and TNF-*α* (Figure 4).

The expression of a number of immunity and inflammatory related genes such as iNOS, COX-2, and IL-6 was modulated by activated NF-*κ*B [25]. Activation of NF-*κ*B involves the phosphorylation and subsequent proteolytic degradation of the inhibitory protein I*κ*B by specific I*κ*B kinases. The free NF-*κ*B (a heterodimer of p50 and p65) then passes into the nucleus, where it binds to NF-*κ*B site in the promoter regions of genes for inflammatory proteins such as cytokines, enzymes, and adhesion molecules [26]. Under inflammatory conditions, inhibitory protein I*κ*B is promptly phosphorylated and degraded from p50 and p65 subunits binding site of NF-*κ*B, and the activated NF-*κ*B subunits migrate to the nucleus. To investigate the possible preventive capability of TIE on NF-*κ*B activation, we studied NF-*κ*B activity, p50/p65 nuclear translocation, and TBK1 expression.

LPS/IFN*γ* stimulation caused the increase of TBK1 expression, activation of NF-*κ*B and induced p50/p65 movement to nucleus, and TIE repressed luciferase intensity of NF-*κ*B promoter in a dose dependent manner (Figure 5(a)). The present study also showed that TIE inhibited LPS/IFN*γ*-induced NF-*κ*B activation through the suppression of TBK1 expression (Figure 5(d)) and on the nuclear translocation of the P50/P65 subunit of NF-*κ*B in RAW264.7 macrophages (Figures 5(b) and 5(c)). So TIE displayed the interference in progress of NF-*κ*B active heterology dimmer heading to the nucleus and the binding capability.

The MAPKs pathway is one of the most ancient and evolutionarily conserved signaling pathways and plays essential regulatory roles in both innate and adaptive immune response [23]. LPS/IFN*γ* stimulation of cytokines production in human monocytes is involved in several intracellular signaling pathways that include three MAPK pathways: ERK1 and ERK2, JNK and p38, and IKK-NF-*κ*B pathway [27]. Thus, we investigated the effect of TIE on activation of ERK1/2, JNK, and p38 in LPS/IFN*γ*-stimulated cells. Our results showed that the phosphorylation of p38 and ERK in response to LPS/IFN*γ* was decreased with TIE treatment, whereas JNK phosphorylation was not affected (Figure 5(e)).

MicroRNAs are short noncoding RNAs that are involved in the epigenetic regulation of cellular processes. The macrophage inflammatory response to LPS stimulation involves the upregulation of miRNAs such as miR155 and miR146 and the downregulation of miR125b. Recently, miR155 has been characterized as a component of the primary macrophage response to different types of inflammatory mediators [28]. Given the powerful role of miR155 in inflammation, it may be an ideal candidate target for anti-inflammatory drug development.

Although results above had been made to identify important and classic genes and pathways involved in the anti-inflammatory effect of TIE, knowledge of noncoding genes such as miRNAs and their contributions is less understood. Our results showed that LPS/IFN*γ* stimulation induced miR155 expression substantially, and TIE reduced LPS plus IFN*γ*-induced miR155 expression (Figure 5(f)). This study highlights a novel mechanism for TIE effects on the inflammatory response via downregulation of miR155 expression.

miR155 is known to be induced downstream of TLR4 signaling. Inhibition of the NF-*κ*B pathway significantly reduced miR155 expression in LPS treated RAW264.7 cells; meanwhile induction of NF-*κ*B activity rapidly leads to increased levels of mature miR155 transcripts. Current study demonstrated that MiR155HG is a direct NF-*κ*B target gene* in vivo* since NF-*κ*B-responsive site exists in the MiR155HG proximal promoter and miR155 is the diced product of the MiR155HG gene [29, 30]. These evidences reflect the importance of the NF-*κ*B pathway in the induction of miR155.

In addition, TIE reduced the nuclear translocation of p65/p50 by inflammatory stimulation, whereas inhibition of NF-*κ*B activation elicited a downregulation of miR155 in LPS treated macrophages, which indicated the important role of the NF-*κ*B pathway in TIE-regulated miR155 expression.

**Figure 6:**
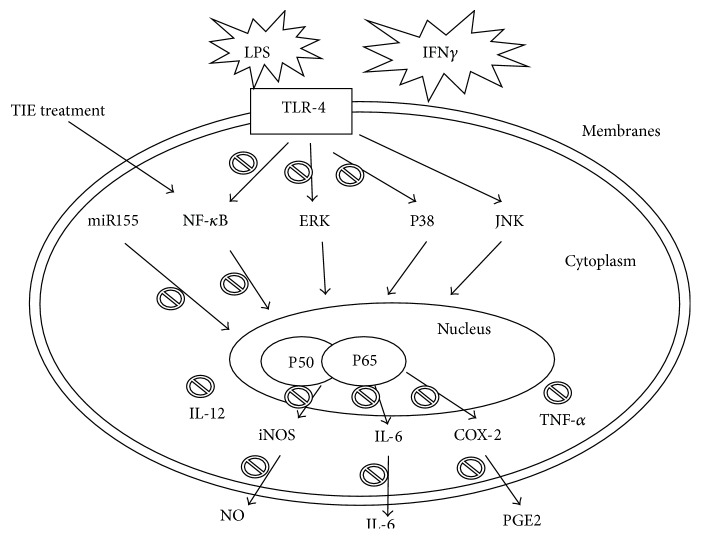
Proposed mechanisms of TIE inhibition of LPS plus IFN*γ* induced inflammation in RAW264.7 cells. TIE inactivates MAPK and NF-*κ*B signaling pathways in addition to inhibiting expression of miR155, which may result from TIE downregulation of iNOS, COX-2, and IL-6 expression and their production. Arrows indicate the main inflammatory pathway activated by LPS stimulation. The prohibition signs indicate the inhibitory effects of TIE.

In conclusion, this is the first investigation of the anti-inflammatory activity of TIE and its functional mechanism in activated macrophages RAW264.7 cells. TIE was found to inhibit the production of NO and PGE2 as well as their upstream enzymes iNOS and COX-2 at protein level as well as secretion and expression of cytokines through LPS/IFN*γ* induced NF-*κ*B/MAPK/miR155 signaling pathways (Figure 6), indicating that TIE has a potential anti-inflammatory application.

## Supplementary Material

The full blot of MAPK showed that TIE markedly inhibited the phosphorylation of ERK and p38 meanwhile had no affect on that of JNK.

## Figures and Tables

**Table 1:** Primer sets for qRT-PCR.

Gene name	Forward primer	Reverse primer
iNOS	GGAGCGAGTTGTGGATTGTC	GTGAGGGCTTGGCTGAGTGAG
COX-2	TGCCTGGTCTGATGATGTATG	AGTAGTCGCACACTCTGTTGT
IL-6	CCACTTCACAAGTCGGAGGCTTA	GTGCATCATCGCTGTTCATACAATC
IL-12p35	ACCTGCTGAAGACCACAGATGACA	TAGCCAGGCAACTCTCGTTCTTGT
IL-12p40	ACCTGTGACACGCCTGAAGAAGAT	TCTTGTGGAGCAGCAGATGTGAGT
TNF-*α*	ATGGGAAGGGAATGAATCCACC	GTCCACATCCTGTAGGGCGTCT
TBK1	ACTGGTGATCTCTATGCTGTCA	TTCTGGAAGTCCATACGCATTG
*β*-actin	GCTACAGCTTCACCACCACAG	GGTCTTTACGGATGTCAACGTC

## Abbreviations

TIE: 1,3,5,7-Tetrahydroxy-8-isoprenylxanthone
LPS: Lipopolysaccharide
IFN*γ*: Interferon-*γ*
NO: Nitric oxide
PGE2: Prostaglandin E2
iNOS: Inducible nitric oxide synthase
COX-2: Cyclooxygenase-2
IL-6: Interleukin-6
IL-12: Interleukin-12
TNF-*α*: Tumor necrosis factor-alpha
TBK1: TANK {TRAF (TNF (tumor necrosis factor) receptor-associated factor) associated NF-*κ*B activator} binding kinase 1
NF-*κ*B: Nuclear factor-kappa B
ERK: Extracellular signal-regulated kinase
JNK: c-Jun N-terminal kinase
MAPK: Mitogen-activated protein kinase
MiR155: MicroRNA 155
ELISA: Enzyme linked immunosorbent assay
TLR4: Toll-like receptor 4.
