# Supplementary figures and images for: Anti-Inflammatory Effect of 1,3,5,7-Tetrahydroxy-8-isoprenylxanthone Isolated from Twigs of Garcinia esculenta on Stimulated Macrophage
Source: Mediators Inflamm. 2015 Oct 11;2015:350564. doi: 10.1155/2015/350564 (PMC4619971; doi:10.1155/2015/350564)

Supplementary data :

Figure 1 Full bolt figure of MAPK pathways effected by TIE

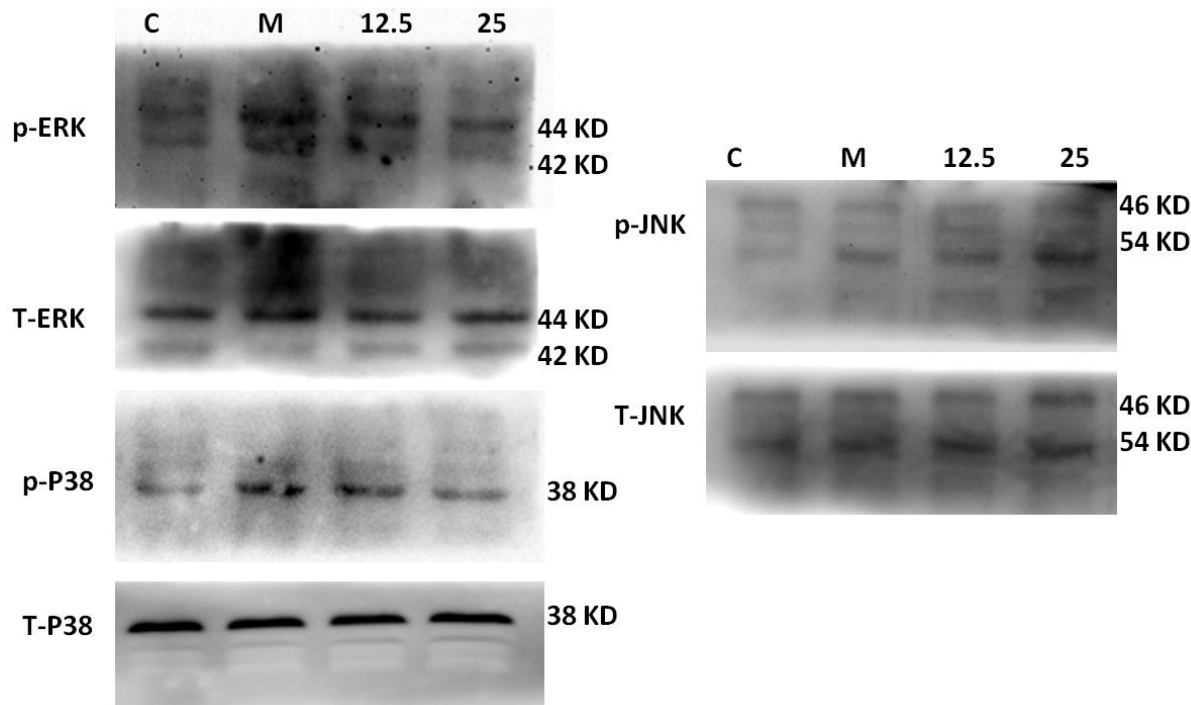

Supplement: Supplementary file 1 — The full blot of MAPK showed that TIE markedly inhibited the phosphorylation of ERK and p38 meanwhile had no affect on that of JNK. [file 350564.f1.pdf]
